# Draft genome sequences of ‘*Candidatus* Liberibacter solanacearum’ haplotype E isolated from carrot psyllids in France

**DOI:** 10.1128/mra.00142-25

**Published:** 2025-10-29

**Authors:** Benoit Remenant, Pascaline Cousseau-Suhard, Aurélie Leroux, Yannick Blanchard, Marianne Loiseau

**Affiliations:** 1 ANSES, Plant Health Laboratory, Bacteriology Virology GMO detection Unit, Angers, France; 2 ANSES, Ploufragan-Plouzané-Niort Laboratory, Viral Genetics and Biosafety Unit, Ploufragan, France; University of Strathclyde, Glasgow, United Kingdom

**Keywords:** draft genome, *Candidatus *Liberibacter solanacearum

## Abstract

Five draft genome sequences of ‘*Candidatus* Liberibacter solanacearum’ haplotype E from five different *Bactericera trigonica* vector insects sampled in France are reported. Each genome has a size of about 1.20 Mbp, a G + C content of 35% and approximately 1,000 predicted coding DNA sequences.

## ANNOUNCEMENT

‘*Candidatus* Liberibacter solanacearum’ (Lso) is a phloem-limited bacterium responsible for vegetative disorders in solanaceous crops in the Americas and New Zealand and in apiaceous crops in Europe. The bacterium is transmitted by host-dependent psyllids (1). Lso is differentiated into haplotypes (2). In France, Lso was first detected in 2012 in the carrot fields (3), where both haplotypes D and E have been identified (4). In the Mediterranean basin, these haplotypes are vectored by *Bactericera trigonica* (1). Since Lso cannot be cultured *in vitro*, genome analysis remains the main tool to characterize this bacterium. Here, we describe the draft genome sequences of LsoE obtained from infected psyllids.

During the summer of 2016, psyllids (*Bactericera trigonica*) from infected carrot fields were collected in the Centre-Val de Loire region, France.

Total DNA was extracted from individual psyllids using the cetyltrimethylammonium bromide (CTAB) method adapted from (5). Each psyllid was ground with 250 µL of 3% CTAB buffer. The suspension was incubated at 65°C for 30 min, then extracted with chloroform:isoamyl alcohol (24:1). DNA was precipitated by adding one volume of cold isopropanol. After centrifugation at 14,000 g for 5 min, the pellet was washed with 70% ethanol, air-dried, and resuspended in 50 µL of sterile water. We determined that the psyllids were contaminated by Lso haplotype E by amplification of the16S rRNA gene, using Lsof/OI2C primers (6, 7), direct sequencing of the PCR products, and comparison with already described haplotypes (8).

For each psyllid, a separate Nextera XT (Illumina Solutions Center Paris, France) library was sequenced with the Novaseq 6000 SP v1.5 kit (2 × 150 cycles; Illumina Solutions Center Paris, France). The number of paired-end reads ranged from 2 to 14 million reads, depending on the sample. Reads were processed using fastp v0.23.1 (9), bases from the 3′ end were trimmed when the mean quality score in a 3-base sliding window was below 20, and reads shorter than 36 nucleotides were deleted. *De novo* assemblies were performed using Spades v3.15.3 (10). Lso contigs were manually selected based on similarity to seven publicly available Lso genomes from BlastN results (v2.13.0) (ZC1 [CP002371.1], R1 [NZ_JNVH00000000.1], HenneA [JQIG00000000.1], NZ1 [JMTK00000000.1], RSTM [NZ_LLVZ01000000.1], FIN111 [NZ_LVWB00000000.1], and ISR100 [NZ_PKRU00000000.2]). Only contigs larger than 1 kb were retained (approximately 50 per genome). Functional automatic annotation of the Lso contigs was performed on the MicroScope platform v3.17.2 (11). Unless otherwise indicated, default parameters were used for softwares.

The main genomic characteristics are provided in Table 1. The average nucleotide identity (orthoANIu) between each pair of assemblies is 99.98% and 100%, indicating high similarity (12, 13).

**TABLE 1:** Carrot psyllid samples and genomes data (annotation was performed on the MicroScope platform v3.17.2)

Strain^*a*^	FR10f	FR12e	FR2f	FR4a	FR9j
City for psyllid collection^*b*^	Corancez	Binas	Le Mée	Levesville La Chenard	Charsonville
Project ID	PRJEB81162	PRJEB81164	PRJEB81165	PRJEB81166	PRJEB81168
GenBank assembly accession number	GCA_964277595.1	GCA_964277575.1	GCA_964277585.1	GCA_964277605.1	GCA_964277615.1
SRA accession number	ERR13953748	ERR13953749	ERR13953745	ERR13953746	ERR13953747
Number of raw reads	4,217,308	5,718,746	13,909,748	2,660,164	4,804,208
Number of contigs	56	54	57	57	56
Draft genome size (bp)	1,196,920	1,194,319	1,195,371	1,199,506	1,194,402
Contig N50	63,434	71,553	65,374	70,388	63,434
Genes	1,248	1,238	1,251	1,244	1,237
Protein coding	1,197	1,187	1,200	1,193	1,186
GC%	34.92	34.91	34.93	34.94	34.92

^*a*^ Correspond to single psyllid sample number from which the bacterium was detected.

^*b*^ All these cities are located in Centre-Val de Loire region.

**Fig 1:**
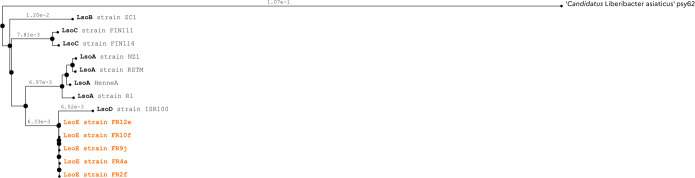
Phylogenetic tree of ‘*Candidatus* Liberibacter solanacearum’ haplotypes rooted with ‘*Candidatus* Liberibacter asiaticus’ strain psy62 constructed with MicroScope platform v3.17.2 (pairwise ANI and Neighbor joining algorithm) (11). The numbers at the tree nodes represent the distance between two nodes of the tree. In orange, genome sequences of LsoE coming from this study compared with the seven publicly available Lso genomes in black (ZC1 [CP002371.1], R1 [NZ_JNVH00000000.1], HenneA [JQIG00000000.1], NZ1 [JMTK00000000.1], RSTM [NZ_LLVZ01000000.1], FIN111 [NZ_LVWB00000000.1], and ISR100 [NZ_PKRU00000000.2]).

Phylogenetic analysis (Fig. 1) confirms the differentiation between haplotype E and the other Lso haplotypes, as well as the genetic proximity between haplotypes E and D, both hosted by Apiaceae and vectored by *B. trigonica*. This phylogeny confirms the previous results obtained with the *rplJ–rplL* gene region (4).

These genome sequences contribute to expanding the available data for this uncultivable bacterium. Coupled with biological information, they will help understand Lso epidemiology, including hosts’ and vector’s specificities across the different geographical areas where it occurs.

## Data Availability

The accession numbers for the genomes of the five strains are shown in Table 1. Draft genome sequences described in this paper are the first versions.
